# Facing off with Scylla and Charybdis: a comparison of scalar, partial, and the novel possibility of approximate measurement invariance

**DOI:** 10.3389/fpsyg.2013.00770

**Published:** 2013-10-23

**Authors:** Rens van de Schoot, Anouck Kluytmans, Lars Tummers, Peter Lugtig, Joop Hox, Bengt Muthén

**Affiliations:** ^1^Department of Methods and Statistics, Faculty of Social Sciences, Utrecht UniversityUtrecht, Netherlands; ^2^Optentia Research Program, Faculty of Humanities, North-West UniversityVanderbijlpark, South Africa; ^3^Department of Public Administration, Erasmus University RotterdamRotterdam, Netherlands; ^4^Center for the Study of Law & Society, University of CaliforniaBerkeley, USA; ^5^Graduate School of Education, University of CaliforniaLos Angeles, CA, USA

**Keywords:** measurement invariance, Bayesian structural equation modeling, Mplus, informative/subjective prior, prior variance

## Abstract

Measurement invariance (MI) is a pre-requisite for comparing latent variable scores across groups. The current paper introduces the concept of approximate MI building on the work of Muthén and Asparouhov and their application of Bayesian Structural Equation Modeling (BSEM) in the software M*plus*. They showed that with BSEM exact zeros constraints can be replaced with approximate zeros to allow for minimal steps away from strict MI, still yielding a well-fitting model. This new opportunity enables researchers to make explicit trade-offs between the degree of MI on the one hand, and the degree of model fit on the other. Throughout the paper we discuss the topic of approximate MI, followed by an empirical illustration where the test for MI fails, but where allowing for approximate MI results in a well-fitting model. Using simulated data, we investigate in which situations approximate MI can be applied and when it leads to unbiased results. Both our empirical illustration and the simulation study show approximate MI outperforms full or partial MI In detecting/recovering the true latent mean difference when there are (many) small differences in the intercepts and factor loadings across groups. In the discussion we provide a step-by-step guide in which situation what type of MI is preferred. Our paper provides a first step in the new research area of (partial) approximate MI and shows that it can be a good alternative when strict MI leads to a badly fitting model and when partial MI cannot be applied.

## Introduction

If scores on a latent variable are to be compared across groups or time in a meaningful way, the underlying measurement model should be equivalent. Measurement invariance (MI) implies that (for continuous observed variables), conditional on the latent trait scores, the covariances and the intercepts are equal across groups (cf. Mellenbergh, 1989). In other words, the relationships between the latent trait scores and the observed variables do not depend on group membership. Studies of so-called “measurement invariance” have often shown that the underlying constructs are, however, *not* equivalent (e.g., Vandenberg and Lance, 2000; Schmitt and Kuljanin, 2008; Millsap, 2011). The current paper discusses approximate MI as a possible solution to these situations, thereby building on the work of Muthén and Asparouhov (2012b, 2013). Muthén and Asparouhov describe a novel method where, using Bayesian structural equation models (BSEM), exact zero constraints can be replaced with approximate zero constraints based on substantive theories. For example, cross-loadings in confirmatory factor analysis are traditionally constrained to be zero, but using the procedure of Muthén and Asparouhov (2012b) these parameters can be estimated with some, as we call it, “wiggle room” (Muthén and Asparouhov, 2012a), implying that very small cross-loadings are allowed. The novel possibility of approximate zero constraints is an interesting alternative to the use of exact zeros which has proven to be unrealistic at times (see for example van Zuiden et al., 2011). The current paper discusses another area where approximate zeros might have an advantage: when full MI across groups is too strict and small differences in factor loadings or intercepts are allowed to make the model fit well. Possibly differences in use of the response scale are described in Morren et al. (2011).

Muthén and Asparouhov (2013) use the BSEM approach as a way to get the non-invariance information as you would get by Maximum Likelihood (ML) modification indices. They propose a two-step procedure where one first uses BSEM's approximate MI analysis to get modification indices and then free those non-invariant parameters in a regular Bayes run as a final, second step. BSEM modification indices are helpful, for example, when having categorical items where no ML modification indices exist, or with a large number of groups. This is often the case in the context of large scale international studies. In the current paper we focus on the benefits or dangers when applying approximate invariance when it is actually applied in a CFA model. As we will show with both an empirical illustration and a simulation study, approximate MI enables the researchers to make explicit trade-offs between the degree of MI on the one hand, and the degree of model fit on the other. However, as our simulation results demonstrate, some bias in the estimated parameters occurs due to the alignment issue (see also Muthén and Asparouhov, 2013), which can be corrected using a method available in Mplus v7.1 (Asparouhov and Muthén, 2013).

In what follows we first illustrate issues with applying MI, followed by an introduction of approximate MI. Thereafter, we provide an empirical illustration where the test for strict MI fails, but where approximate MI results in a well-fitting model. Then, with a simulation study, we investigate whether approximate MI can lead to unbiased estimates for differences in latent scores across groups. Thereafter, we introduce the correction method and show its influence on the parameters in our simulation study. We conclude with a discussion and practical recommendations for scholars who aim to meaningfully compare scores on latent variables. Note that the application of approximate MI in the current paper is limited to situations with a small number of groups, continuous variables, and “almost” invariant models. For a more general approach see Muthén and Asparouhov (2013).

## The issue of approximate measurement invariance: Scylla or Charybdis

Questionnaires are often used to assess latent constructs, such as human attitudes and behavior, with the goal to compare groups. For such a comparison to be valid MI should apply, see (Millsap, 2011) or Vandenberg and Lance (2000) for a comprehensive overview on possible methods testing MI. That is, a questionnaire should measure identical constructs with the same factor structure across different groups. Stated differently, factor loadings, intercepts, and residual variances should be identical to get the label “full measurement invariance.” If one wants to compare latent means the intercepts are of major importance and therefore, we focus on the intercepts.

Van de Schoot et al. (2012) stated that “When MI does not hold, groups or subjects […] respond differently to the items and as a consequence factor means cannot reasonably be compared” (p. 487). This statement refers to a potential bias in the latent mean comparison when full MI is assumed, but not supported by the data, or when MI is not assumed and the latent means are (incorrectly) compared. In order to meaningfully compare latent means across groups, at least the factor loadings and intercepts should be equal; this is the situation of scalar invariance (Vandenberg and Lance, 2000). Henceforth, when (full) MI is used we refer to scalar invariance. After testing for scalar MI it might be that such a model does not fit the data. What to do in such a situation? One solution is to allow for partial MI. Steenkamp and Baumgartner (1998) suggested that as long as at least two of the factor loadings and intercepts are constrained to be equal across groups or time, the difference in the latent mean between the groups is unbiased (see also Steinmetz, 2013). However, this procedure has been debated much (Vandenberg, 2002), for example how to choose the reference category (Rensvold and Cheung, 2001). At least partial invariance for the factor loadings before one can proceed to test invariance of the intercepts (Steenkamp and Baumgartner, 1998). This paper focuses on comparison of latent means, so we present approximate MI in the context of the intercepts.

To sum up, if MI is used to either see if measurement instruments are equivalent across populations, or to compare the latent means to each other, possible outcomes of MI are:

1. (full or) scalar MI, where all intercepts are constrained to be equal across groups.
2. partial MI, where some of the intercepts between groups are allowed to be freely estimated, while others are held constant (see e.g., Steenkamp and Baumgartner, 1998; Steinmetz, 2013); or
3. No invariance, where all intercepts between groups are freely estimated, because such a model fits the data best. Consequently, the questionnaire cannot be used for comparing groups.
  In the current paper we add a fourth option, initiated by Muthén and Asparouhov (2012b, 2013) and introduced in more detail below:
4. Approximate MI, a Bayesian solution allowing for some wiggle room for the intercept differences between groups, where the wiggle room is determined by the degree of precision of the prior.

Metaphorically speaking, in testing for MI one has to choose between Scylla and Charybdis, two mythical Greek sea monsters^1^. In the current paper we apply this metaphor to the procedure of testing for MI. On the one hand, there is the six-headed sea monster Scylla, who metaphorically represents imposing full MI on the model with as a result that the model fit indices indicate a bad fit to the data. On the other hand, however, we could fall victim to Charybdis if we release the constraints. By not imposing MI, our model will fit the data, but it will be impossible to compare groups. This paper illustrates the third option, using approximate MI, which could turn out to be the way to escape both threats.

**Figure 1:**
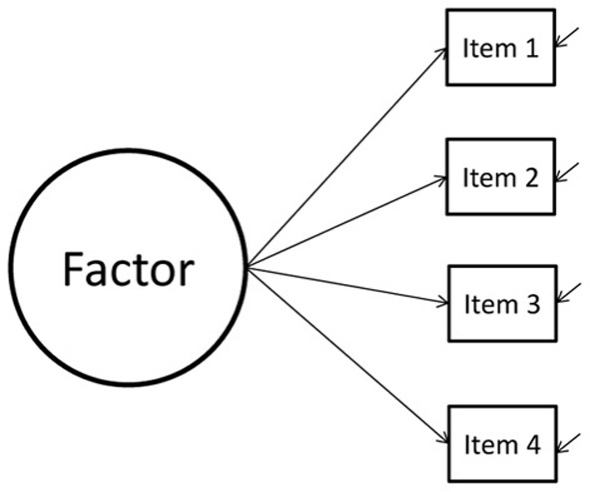
**A hypothetical model**.

**Figure 2:**
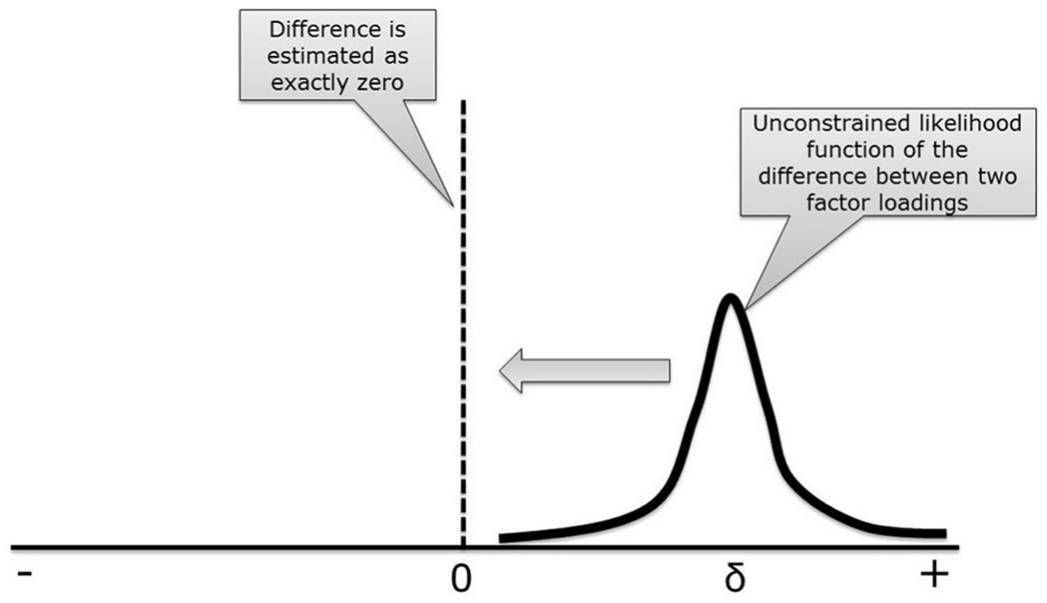
**The influence of applying MI while the difference between factor loading is clearly not zero**.

Consider a CFA model with two groups, see Figure 1. Suppose the difference between the intercepts of item 1 is 0.10. Now, we impose MI on this model, by constraining the two intercepts to be equal. As a result, the difference between both will be exactly zero, that is, we are imposing a difference of zero on the parameter estimates for the intercepts. In Figure 2 the likelihood function (which is a function of the distribution of the data) is shown for the difference between both intercepts, which is denoted by δ. In this case there is a small difference in the intercepts between both groups. When applying MI, the difference is forced to be zero (δ = 0). By doing this, we have established MI and we are allowed to compare the latent factor means between the two groups. However, the estimated intercepts no longer resembles their unconstrained counterparts. Stated differently, δ is forced to be zero, whereas in the data δ > 0. The discrepancy between δ in our model and δ in the data will probably result in poor model fit. A bad model fit means we have to reject our model and cannot interpret our model parameters.

Meanwhile, on the other side of the narrow channel between Italy and Sicily, Charybdis lurks, forced to live in a cave beneath the sea causing whirlpools. If we would analyze our hypothetical model without any constraints on the intercepts the model will fit the data. As a consequence, however, we are lost in the whirlpools caused by the furious Charybdis, because we can no longer compare the latent means due to different intercepts across the groups.

There we are, trapped between Scylla and Charybdis, and are forced to choose between either a model with MI and a terrible fit to the data, or a well-fitting model that we cannot use for comparing the latent means across groups. However, just like Odysseus, we believe we can pass in safety through the narrow channel. One passage may be provided by imposing partial MI allowing for one or two differences. Partial MI seems attractive when relatively large differences (δ >> 0) exist for one or only a few items. However, when differences are small and occur for multiple items in a factor analysis, partial MI is not able to provide a safe passage and a*pproximate* MI offers an attractive alternative. With approximate MI, instead of forcing intercepts to be exactly equal across groups, see Figure 2, a substantive prior distribution is used to bring the parameters close to one another while allowing for some wiggle room. Such a model falls in between full and no MI, which could mean that we can still compare the means (as MI holds approximately) while the model also fits well, allowing an escape from Scylla and Charybdis. But how does this work?

### Using Bayesian priors on intercept differences

To estimate a model with approximate MI we need Bayesian statistics, which has been discussed in many papers and textbooks (see, among others, Kruschke et al., 2012; Van de Schoot et al., 2013). There are three essential ingredients underlying Bayesian statistics. The first ingredient is prior distributions which represent background knowledge about the parameters of a model; for example that the difference between two intercepts is close to zero. Second, there is the likelihood function of the data containing the information about the parameters from the data. Thirdly, both prior and likelihood are summarized by the so-called posterior distribution, which is a compromise of the prior knowledge and the likelihood function. Stated otherwise, the posterior distribution contains one's updated knowledge balancing prior knowledge with observed data.

**Figure 3:**
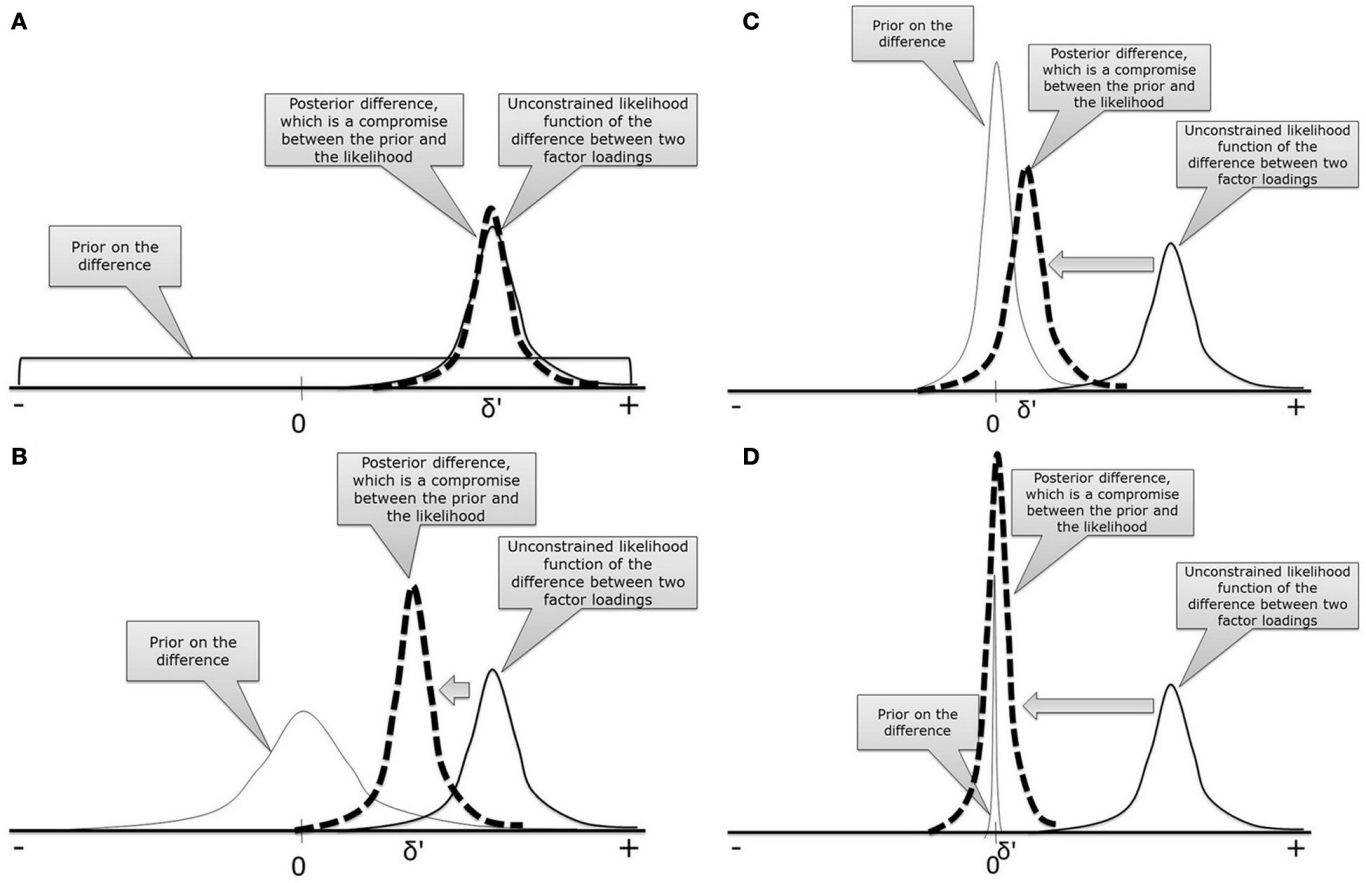
**Four different prior distributions to demonstrate the influence of the prior on the posterior parameter estimates. (A)** Uninformative prior; **(B)** Wide normal prior; **(C)** Narrow normal prior; **(D)** Highly peaked prior.

The crucial ingredient of Bayesian statistics is the specification of the prior distribution. In Figure 3, four different priors are specified and combined with the likelihood function of the difference between two intercepts which is denoted by δ. When combining prior and likelihood, the posterior difference score is obtained, denoted by δ′. Figure 3A displays a flat uninformative prior for the difference between the two intercepts. Because such a prior does not contain any information, the posterior estimate for the difference will not be influenced and the results are similar to a model without MI, that is δ = δ′. If, for example, a normal prior distribution is used, see Figure 3B, the posterior estimate for the difference, δ′, will be slightly pulled toward the mean of the prior, in this case zero. If we decrease the prior variance, see Figure 3C, the posterior difference comes closer to zero. If the prior variance is very small, the posterior difference will approximate zero, δ′ ~ 0, and we establish approximate MI allowing for some wiggle room. To get back to our metaphor: if a small difference between the intercepts is allowed, we can escape Charybdis because the difference between intercepts is smaller than in the unconstrained model, Figure 3A. We also escape Scylla, because a model with some wiggle room is less restrictive than full MI and will therefore, still fit the data acceptably well, Figure 2. In conclusion, approximate MI finds a compromise between zero and no constraints, through which both model fit and latent mean comparison can be established.

Approximate MI is expected to be especially useful when there are many small deviations from strict MI (De Boeck, 2008; Muthén and Asparouhov, 2013). In the current paper we focus on studying the differences between strict, partial and approximate MI in a set of populations. In the current paper we assume that the main goal of applying MI is to compare latent means and, therefore, focus on the potential bias in the latent mean comparison when different degrees of MI are applied. There are two indicators to keep in mind: (1) model fit and (2) a small enough difference between either factor loadings or intercepts.

## Empirical illustration

### Introduction

The empirical illustration looks at the experiences of psychologists (group 1) and psychiatrists (group 2) with a new policy in Dutch mental healthcare: Diagnosis Related Groups (DRGs; Tummers et al., 2012). Diagnosis Related Groups were introduced in January 2008 and were part of a process to convert the Dutch healthcare system into one based on a regulated market. The DRG policy differs significantly from the prior method in which each medical action resulted in a financial claim, a so-called fee-for-service system. Before 2008, the number of sessions a professional had with a patient related directly to the amount of money claimed from the health insurer. According to some standpoints, this could lead to inefficiencies (Busse et al., 2011). The DRG policy changed the situation by stipulating a standard rate for each disorder. For instance, for a mild depression, the mental healthcare professional gets a standard rate for treating the patient (direct and indirect time) between 250–800 min.

Psychologists and psychiatrists had to implement these DRGs, and we will investigate their willingness to do so. This is important, as many of them opposed the DRG policy, set up websites agitating against it, or even in a few cases quit their jobs (Palm et al., 2008). The following quote of a healthcare professional [cited in Tummers (2012): 516], illustrates their point of view:

“We experience the DRG policy as a disaster. I concentrate as much as possible on treating my own patients, in order to derive some satisfaction from my work.”

Furthermore, psychiatrists were far more resistant than psychologists. One of the reasons was that especially psychiatrists considered the DRGs as a threat to their autonomy (Smullen, 2013). It is important to analyze the difference between the two groups, in order to provide guidance to policy makers in their attempts to adapt the policy and increase the satisfaction of professional health workers. We would expect minor violations of MI given that the both groups of professionals were expected to be quite negative about the specific policy and also have slightly different attributes to the concepts used in the questionnaires because of their professional training and working environment (see for instance Palm et al., 2008; Neukrug, 2011; Smullen, 2013).

### Methods

The sampling frame consisted of 5199 professionals, all members of the two main nationwide mental healthcare associations: the Dutch Association of Psychologists (NIP) and the Netherlands Association for Psychiatry (NVvP), who would, in principle, all of them be required to work with the DRG policy. Using an email and two reminders, 1307 questionnaires were returned; a response rate of 25% with 1074 valid cases for the specific scale we used. Despite the select sample the demographical composition of the respondent group was representative for the Dutch population of mental healthcare professionals (Palm et al., 2008).

Willingness to implement the DRG policy was measured using a validated four-item scale developed by Metselaar (1997), which is based on the notion of “intention to act” in the theory of planned behavior (Ajzen, 1991). The items use five-point Likert-scale response categories (strongly disagree, disagree, neutral, agree, and strongly agree). The items use templates in which one can specify the change being assessed, for example, the item “I intend to make time to implement the change” was changed into “I intend to make time to implement the DRG-policy.” All item descriptions, its means, variances, and correlations are included in Table 1 and the data and all syntax files are available on the website of the first author.

**Table 1:** **Correlation matrix for Psychologists (*n* = 570) and Psychiatrists (*n* = 504) with the means (variances) on the diagonal**.

	**1**	**2**	**3**	**4**
1. I intend to try to convince employees of the benefits the DRG-policy	2.023 (0.727)/1.831 (0.730)			
2. I intend to put effort into achieving the goals of the DRG-policy	0.589/0.549	2.651 (1.040)/2.414 (1.137)		
3. I intend to reduce resistance among employees regarding the DRG-policy	0.727/0.737	0.616/0.599	2.353 (0.763)/2.186 (0.950)	
4. I intend to make time to implement the DRG-policy	0.451/0.470	0.442/0.492	0.483/0.514	2.795 (0.939)/2.472 (1.091)

### Results

If we want to compare psychologists and psychiatrists on the willingness to implement DRGs, we could simply compare the mean scores based on the four items. It appeared that, using a *T*-test in SPSS, psychiatrists (*M* = 2.23; *SD* = 0.81; *n* = 504) indeed scored significantly lower compared to psychologists (*M* = 2.46; *SD* = 0.76; *n* = 570; *M*_*dif*_ = 0.23; *t* = 4.83; *p* < 0.001). However, by using the mean score we assume that each item reflects the underlying construct in the same way and, even more importantly, that there is no measurement bias (Steinmetz, 2013). To accommodate these unwanted side-effects we conducted a series of confirmatory factor analyses (CFA) using the software M*plus* v7 (Muthén and Muthén, 1998-2012). The data and all syntax files are available as supplementary materials.

In the first model, a 2-group configural model, because of the (slightly) non-normal distributed items estimated with ML estimator with robust standard errors (i.e., MLR), we allowed the factor loadings and intercepts to vary across groups resulted in a well-fitting model (χ^2^ = 12.982; *df* = 4; *p* = 0.011; RMSEA = 0.065; CFI = 0.992; TLI = 0.976) with standardized factor loadings ranging between 0.56–0.87. We tested for MI using the new option in Mplus v7.11 ANALYSIS: MODEL = CONFIGURAL METRIC SCALAR. A model forcing scalar MI, i.e., factor loadings and intercepts were constrained across groups, appeared to fit the data well (χ^2^ = 32.032; *df* = 10; *p* < 0.001; RMSEA = 0.064; CFI = 0.980; TLI = 0.976), but not better compared to the configural model (Δχ^2^ = 19.479; Δ*df* = 6; *p* = 0.003). Also the metric model, where only the factor loadings were held equal across groups, fitted the data (χ^2^ = 18.605; *df* = 7; *p* = 0.009; RMSEA = 0.056; CFI = 0.990; TLI = 0.982) and not any worse compared to the configural model (Δχ^2^ = 5.019; Δ*df* = 3; *p* = 0.170). We also ran a comparison between the scalar and metric model and it appeared that the scalar model fits the data worse compared to the metric model (Δχ^2^ = 13.988; Δ*df* = 3; *p* = 0.003). According to most fit indices (e.g., χ^2^ not significantly worse than the configural model, but significantly better than the scalar model) the best model appeared to be the metric model where the factor loadings are constrained while the intercepts are allowed to differ across groups.

A solution offered by, for example Byrne et al. (1989; see also Steenkamp and Baumgartner, 1998), is to apply partial MI. To establish partial invariance, one studies the size of the unconstrained loadings and/or intercepts, and constrains all loadings and intercepts except for the one loading/intercept with the largest unstandardized difference, which is released. It appeared that psychiatrists have lower intercepts than the psychologists, with the differences being 0.193, 0.235, 0.167, and 0.324, respectively. We applied partial MI, that is, constraining the intercepts of items 1 and 3 while releasing the constraints on intercepts 2 and 4 (χ^2^ = 20.271; *df* = 8; *p* = 0.009; RMSEA = 0.053; CFI = 0.989; TLI = 0.983). Using the procedure described on the website of Mplus to compute MLR chi-square difference testing, it appeared that the partial model did not result in a better fit compared to the metric model (Δχ^2^ = 1.502; Δ*df* = 1; *p* = 0.203), but better compared to the scalar model (Δχ^2^ = 12.313; Δ*df* = 2; *p* = 0.002).

We re-analyzed the two models, constrained and unconstrained intercepts, using the ML and Bayesian estimator using the default prior settings [i.e., normal prior distributions for the intercepts and factor loadings with a prior mean of zero and a prior variance of 10^10^, and an inverse gamma distribution for the (residual) variance terms with hyperparameters −1 and zero], but with a stricter cut-off value for convergence to reduce any bias caused by precision [i.e., Chains = 8, Bconvergence = 0.01 and Biterations(20000)]. Table 2 shows the results for the intercepts, the difference between the intercepts, and the Bayesian model fit information. These results show that a model with strict MI assumed does not fit the data. This is shown by the fact that (1) the posterior predictive *p*-value is significant, and (2) the 95% CI of the replicated Chi Square values does not include zero. Hence, the model without MI does fit the data, but we are not allowed to compare the latent means between psychiatrists and psychologist.

**Table 2:** **The results for the intercepts of the latent variable *Willingness to Implement DRGs***.

		**Model A**		**Model B**		**Model C**		**Model D**		**Model E**		**Model F**		**Model G**	
		**Measurement invariance**		**No constraints on the intercepts**		**Approximate MI σ^2^ = 0.50**		**Approximate MI σ^2^ = 0.05**		**Approximate MI σ^2^ = 0.01**		**Approximate MI σ^2^ = 0.005**		**Approximate MI σ^2^ = 0.0005**	
		***ν* (*SE*)**	**95% *CI***	***ν* (*SE*)**	**95% *CI***	***ν* (*SE*)**	**95% *CI***	***ν* (*SE*)**	**95% *CI***	***ν* (*SE*)**	**95% *CI***	***ν* (*SE*)**	**95% *CI***	***ν* (*SE*)**	**95% *CI***
Intercepts group = psychologists	Item 1	2.022 (0.032)	1.961–2.085	2.022 (0.035)	1.955–2.091	2.020 (0.034)	1.954–2.088	2.006 (0.034)	1.943–2.072	1.979 (0.034)	1.957–2.090	1.961 (0.030)	1.904–2.021	1.935 (0.027)	1.885–1.990
	Item 2	2.634 (0.037)	2.563–2.709	2.650 (0.042)	2.569–2.733	2.647 (0.042)	2.565–2.731	2.631 (0.041)	2.550–2.712	2.597 (0.041)	2.569–2.729	2.577 (0.037)	2.506–2.649	2.545 (0.033)	2.483–2.610
	Item 3	2.372 (0.034)	2.308–2.440	2.352 (0.036)	2.281–2.425	2.349 (0.037)	2.278–2.420	2.334 (0.036)	2.264–2.402	2.305 (0.035)	2.285–2.424	2.286 (0.032)	2.224–2.349	2.269 (0.029)	2.212–2.723
	Item 4	2.724 (0.035)	2.657 (2.792)	2.795 (0.041)	2.713–2.876	2.792 (0.041)	2.713–2.868	2.775 (0.040)	2.697–2.851	2.739 (0.040)	2.704–2.859	2.716 (0.036)	2.642–2.783	2.660 (0.032)	2.596–2.723
Intercepts group = psychiatrists	Item 1	2.022 (0.032)	1.961–2.085	1.830 (0.039)	1.757–1.908	1.831 (0.038)	1.758–1.905	1.847 (0.037)	1.771–1.919	1.881 (0.062)	1.917–2.162	1.896 (0.032)	1.836–1.961	1.925 (0.027)	1.873–1.978
	Item 2	2.634 (0.037)	2.563–2.709	2.413 (0.048)	2.323–2.508	2.415 (0.048)	2.323–2.509	2.434 (0.046)	2.346–2.526	2.477 (0.066)	2.503–2.765	2.496 (0.040)	2.420–2.578	2.533 (0.034)	2.468–2.600
	Item 3	2.372 (0.034)	2.308–2.440	2.185 (0.043)	2.103–2.270	2.186 (0.043)	2.103–2.269	2.204 (0.041)	2.124–2.285	2.241 (0.069)	2.287–2.562	2.257 (0.036)	2.188–2.329	2.275 (0.030)	2.217–2.336
	Item 4	2.724 (0.035)	2.657 (2.792)	2.472 (0.046)	2.383–2.562	2.472 (0.046)	2.382–2.563	2.492 (0.045)	2.404–2.581	2.539 (0.058)	2.549–2.777	2.564 (0.039)	2.489–2.643	2.629 (0.033)	2.566–2.695
Difference in intercept	Item 1	0		0.192		0.189		0.159		0.098		0.065		0.010	
	Item 2	0		0.237		0.232		0.197		0.120		0.081		0.012	
	Item 3	0		0.167		0.163		0.130		0.064		0.029		−0.006	
	Item 4	0		0.323		0.320		0.283		0.200		0.152		0.031	
Model fit	95% CI for the difference between the observed and the replicated χ^2^	5.128 44.154		−4.164 34.566		−5.516–40.199		−4.369–38.364		−5.543–39.921		3.573–48.979		18.248–60.600	
	Posterior predictive *p*-value	0.008		0.067		0.057		0.062		0.031		0.012		0.000	

The new option is to use approximate MI. Using Bayesian statistics parameters can be restricted by specifying a prior distribution. We would like the difference between the intercepts to approximate zero, but to allow for some wiggle room (prior variance) to maintain a fitting model. That is, the difference in an intercept between the two groups is allowed to exist, but is restricted to be very small, which is established by specifying a specific prior distribution of that difference. We used the new DIFF option available in M*plus* v7 within the MODEL PRIOR part of the syntax where subjective priors can be specified. The full syntax is shown in the Appendix A, but the most important part is:


MODEL:[Veran1- Veran4] (nu#_1 - nu#_4);
MODEL PRIOR: DO(1,4) DIFF(nu1_#-nu2_#) N(0,0.50);


where (nu#_1 - nu#_4) defines labels for the four intercepts. Because we used #,the labels are automatically specified for both groups separately. The DO(1,4) option is a loop statement telling M*plus* to apply the function which comes after the DO statement for items 1 through 4: #=1 to #=4. The DIFF statement refers to the difference between the first intercept of the psychiatrists, for example nu1_1, and the same intercept for psychologists, for example nu1_2. Because we used the DO option this is automatically repeated for all four intercepts. Furthermore, ~ N(0, 0.50) indicates the intercept differences between groups to be normally distributed (N) with mean 0 and prior variance of 0.5 for all pairs of items. Note that we parametrized the model by forcing both latent means to zero and the variance to one.

The results for this specific model are shown in Table 2 in the column labeled Model C. We varied the prior variance by using σ^2^ = 0.05 (Model D), σ^2^ = 0.01 (Model E), σ^2^ = 0.005 (Model F), and σ^2^ = 0.0005 (Model G). In Model C, with a large prior variance, the difference between intercepts appeared not to be smaller compared to the unconstrained Model B. In Model D, however, the influence of the prior specification can be observed: the difference between intercepts becomes smaller. In Model E the intercepts are even closer, in Model F they are very close and in Model G they are almost similar. However, the latter two models do not fit the data very well; i.e., the 95% CI for the difference between the observed and the replicated χ^2^ does not include zero and the *ppp*-value (i.e., posterior predictive *p*-value) is < 0.01. In sum, allowing for a prior variance of 0.01 between the intercepts, as is the case in Model E, resulted in an acceptable model fit. Also, the confidence interval of Δχ^2^ does include zero. However, the posterior predictive *p*-value is significant, and preferably should be closer to 0.50. A larger reduction, which would be a model closer to scalar invariance, did not fit the data.

To summarize, we have established MI using the newly available approximate MI method. Now, we can finally conclude that psychiatrists score significantly lower on the willingness to implement DRGs than psychologists. The mean difference equals 0.21 (*p* < 0.001), which would indeed be somewhat different had we used full MI (*M*_*dif*_ = 0.19) or an unconstrained model (*M*_*dif* = 0.14)_.

However, little is known about the bias of parameters as a result of approximate MI. Therefore, in the next section we will conduct a simulation study to find out if we are truly allowed to interpret the mean difference of the latent mean between groups if we apply approximate MI.

## Simulation study

### Method

To investigate the possible bias in the comparison of latent means as a result of applying the approximate MI model we performed a simulation study. Seven populations were specified from which we obtained 1000 datasets each. The difference in intercepts between both groups varied across these seven populations, see Table 3. All other parameters were kept constant across populations; see Appendix B for the syntax and model specifications. Most importantly, the mean of the latent variable in group 1 was set to 0 and in group 2 to 0.5. Both latent factors were specified to have a population variance of 1. All items are standardized making the latent mean difference between the two groups of 0.5 a medium effect size (Cohen, 1992). The sample size per group was specified as being 500.

**Table 3:** **Population values for the intercepts**.

	**Intercepts**							
	**Item 1**		**Item 2**		**Item 3**		**Item 4**	
	**Group 1**	**Group 2**	**Group 1**	**Group 2**	**Group 1**	**Group 2**	**Group 1**	**Group 2**
Population 1	0	0	0	0	0	0	0	0
Population 2	0	0	0	0	−0.01	0.01	−0.01	0.01
Population 3	0	0	0	0	−0.1	0.1	−0.1	0.1
Population 4	0	0	0	0	−0.5	0.5	−0.5	0.5
Population 5	−0.01	0.01	−0.01	0.01	−0.01	0.01	−0.01	0.01
Population 6	−0.1	0.1	−0.1	0.1	−0.1	0.1	−0.1	0.1
Population 7	−0.5	0.5	−0.5	0.5	−0.5	0.5	−0.5	0.5

The seven populations described in Table 3 were confronted with a set of models:

– Model 1: *scalar* MI is applied to the intercepts *and* factor loadings. Results were obtained with ESTIMATOR = ML and with ESTIMATOR = BAYES. For the latter we used BCONVERGENCE = 0.01, BITERATIONS = (5000), and the default priors [i.e., normal prior distributions for the intercepts and factor loadings with a prior mean of zero and a prior variance of 10^10^, and an inverse gamma distribution for the (residual) variance terms with hyperparameters −1 and zero].
– Model 2: *partial* MI is applied to those intercepts that are not similar in the population. For population 1 no partial MI can be applied, since all intercepts are similar in the population, for populations 2–4 partial MI is applied to the intercepts of items 3 and 4, and for populations 5–7 partial MI is applied to all intercepts. Note that the factor loadings are held equal across groups. Results were obtained with ESTIMATOR = ML and with ESTIMATOR = BAYES. For the latter, we used BCONVERGENCE = 0.01, BITERATIONS = (5000), and the default priors.
– Model 3: *approximate* MI is applied only to the intercepts. We varied the prior variance: σ^2^ = 0.5 (Model 3a), σ^2^ = 0.05 (Model 3b), σ^2^ = 0.01 (Model 3c), and σ^2^ = 0.005 (Model 3d). For all other parameters we used the default prior settings.
– Model 4: *partial* approximate MI, where wiggle room is applied only to those intercepts that are not equal in the population; populations 2–4. We varied the amount of prior variance: σ^2^ = 0.5 (Model 4a), σ^2^ = 0.05 (Model 4b), σ^2^ = 0.01 (Model 4c), and σ^2^ = 0.005 (Model 4d).

The simulated differences in intercepts may cause an alignment issue, i.e., a biased estimate of the latent mean difference across groups, which will be discussed in more details in the next section. Because researchers usually wish to compare latent means across groups, we focus on whether or not the estimated difference in latent means is biased. We focused on four outcome criteria that might indicate the degree to which the mean difference is biased:

1. the empricial standard deviation of the 1000 estimated mean differences, which should be <0.10.
2. the relative mean bias defined as ((*M* − 0.5)/0.5)^*^100, where M is the average mean obtained from the simulation study. We used a cut-off value of <10% as a criterion, as suggested by Hoogland and Boomsma (1998) for “reasonable” accuracy.
3. The proportion of replications with a *ppp*-value smaller than pre-specified cut-off values. 95% coverage of the population value and its 95% significance.

Note that, concerning (3), the *ppp*-value, which defined as the proportion of chi-square values obtained in the simulated data that exceed that of the actual data and *ppp*-values around 0.50 indicate a well-fitting model.

To determine whether the simulation results resemble a good model fit, the proportion of replications where the critical value of 0.05 is exceeded should be close to 0.05, as *p*-values are expected to be uniformly distributed. The 95% coverage is defined as the percentage of replications for which the 95% CI included the population value of ΔM = 0.5. The significance criterion was defined as the percentage of datasets for which the 95% CI did not include zero, i.e., the percentage of datasets for which we would have concluded that ΔM is larger than zero in the population, which it was for all populations.

### Results

Table 4 provides the results for Model 1 and 2 with ML and Bayesian estimation, Table 5 provides the results for Models 3a–3d and Table 6 provides the results for Models 4a–4d. We will first discuss the results row wise, i.e., per model, followed by a column wise comparison, i.e., per population.

**Table 4:** **Simulation results for Model 1 and 2**.

**Model**	**Outcome**	**Population 1:**		**Population 2:**		**Population 3:**		**Population 4:**		**Population 5:**		**Population 6:**		**Population 7:**	
		**No differences**		**2 items with small differences**		**2 items with moderate differences**		**2 items with large differences**		**4 items with small differences**		**4 items with moderate differences**		**4 items with large differences**	
		**ML**	**Bayes**	**ML**	**Bayes**	**ML**	**Bayes**	**ML**	**Bayes**	**ML**	**Bayes**	**ML**	**Bayes**	**ML**	**Bayes**
#1 Full measurement invariance	Estimated ΔM and *SE*	0.4995	0.4976	0.5117	0.5097	0.6501	0.6488	2.3699	2.3366	0.5362	0.5341	0.8786	0.8768	2.6222	2.6619
		(0.0917)	(0.0980)	(0.0920)	(0.0985)	(0.0976)	(0.1061)	(0.2192)	(0.2224)	(0.0923)	(0.0989)	(0.0995)	(0.1086)	(0.1679)	(0.1837)
	Convergence	100%	100%	100%	100%	100%	100%	100%	100%	100%	100%	100%	100%	100%	100%
	Relative bias ΔM%	−0.1	−0.48	2.34	1.94	30.02	29.76	373.98	367.32	7.24	6.82	75.72	75.72	424.44	432.38
	95% coverage	95.9%	95.1%	96.1%	94%	65.3%	56.9%	0%	0%	93.9%	91.2%	2.2%	1.5%	0%	0%
	95% significance	100%	100%	100%	100%	100%	100%	100%	100%	100%	100%	100%	100%	100%	100%
#2 Partial measurement invariance	Estimated ΔM and *SE*	0.4990	0.4863	0.4990	0.4863	0.4990	0.4863	0.4990	0.4863	0.5298	0.5169	0.7904	0.8079	2.0493	1.9829
		(0.0979)	(0.0988)	(0.0979)	(0.0988)	(0.0979)	(0.0988)	(0.0979)	(0.0988)	(0.0984)	(0.0992)	(0.103)	(0.104)	(0.148)	(0.123)
	Convergence	100%	100%	100%	100%	100%	100%	100%	100%	100%	100%	100%	100%	100%	100%
	Relative bias ΔM%	−0.2	−2.74	−0.2	−2.74	−0.2	−2.74	−0.2	−2.74	5.96	3.38	58.08	61.58	309.86	296.58
	95% coverage	94.8%	94.3%	94.8%	94.3%	94.8%	94.3%	94.8%	94.3%	94.6%	94.4%	17.3%	13.9%	0%	0%
	95% significance	99.9%	99.9%	99.9%	99.9%	99.9%	99.9%	99.9%	99.9%	100%	100%	100%	100%	100%	100%

**Table 5:** **Simulation results for Model 3**.

**Model**	**Outcome**	**Population 1:**	**Population 2:**	**Population 3:**	**Population 4:**	**Population 5:**	**Population 6:**	**Population 7:**
		**No differences**	**2 items with small differences**	**2 items with moderate differences**	**2 items with large differences**	**4 items with small differences**	**4 items with moderate differences**	**4 items with large differences**
#3a N~(0, 0.5)	Estimated ΔM and *SE*	0.0404	0.8537	0.6417	1.1153	0.8779	0.9018	2.3347
		(0.5161)	(0.5923)	(0.6627)	(0.7033)	(0.5924)	(0.6975)	(0.7101)
	Convergence	100%	100%	100%	100%	100%	99.4%	99.8%
	Relative bias ΔM(%)	−91.92	70.74	28.34	123.06	75.58	80.36	366.94
	95% coverage	92.9%	100%	100%	100%	100%	100%	0%
	95% significance	0%	0.1%	0%	2.1%	0.1%	0%	100%
#3b N~(0, 0.05)	Estimated ΔM and *SE*	0.4143	0.5378	0.6125	1.1672	0.5622	0.8560	2.3644
		(0.2294)	(0.2239)	(0.2393)	(0.2612)	(0.2240)	(0.2409)	(0.2709)
	Convergence	100%	100%	100%	100%	100%	100%	100%
	Relative bias ΔM(%)	−17.14	7.56	22.5	133.44	12.44	71.2	372.88
	95% coverage	100%	100%	99.9%	7.3%	99.9%	87.6%	0%
	95% significance	45.9%	89.5%	97.1%	100%	94.2%	100%	100%
#3c N~(0, 0.01)	Estimated ΔM and *SE*	0.4554	0.5124	0.6167	1.6506	0.5368	0.8596	2.4984
		(0.1246)	(0.1352)	(0.1320)	(0.2169)	(0.1353)	(0.1368)	(0.2011)
	Convergence	100%	100%	100%	100%	100%	100%	100%
	Relative bias ΔM(%)	−8.92	2.48	23.34	230.12	7.36	71.92	399.68
	95% coverage	98.2%	99.7%	94.7%	0%	99.7%	17.2%	0%
	95% significance	99.4%	99.8%	100%	100%	99.9%	100%	100%
#3d N~(0, 0.005)	Estimated ΔM and *SE*	0.4671	0.5084	0.6218	1.9494	0.5328	0.8611	2.5453
		(0.1072)	(0.1173)	(0.1122)	(0.2205)	(0.1175)	(0.1142)	(0.1900)
	Convergence	100%	100%	100%	100%	100%	100%	100%
	Relative bias ΔM(%)	−6.58	1.68	24.36	289.88	6.56	72.22	409.06
	95% coverage	97.3%	98.9%	86.9%	0%	98.6%	7.7%	0%
	95% significance	99.8%	100%	100%	100%	100%	100%	100%

**Table 6:** **Simulation results for Model 4**.

**Model**	**Outcome**	**Population 2:**	**Population 3:**	**Population 4:**
		**2 items with small differences**	**2 items with moderate differences**	**2 items with large differences**
#4a (N~(0, 0.5))	Estimated ΔM and *SE*	0.4926 (0.0993)	0.4939 (0.0994)	0.4998 (0.1000)
	Convergence	100%	100%	100%
	Relative bias ΔM(%)	−1.48	−1.22	−0.04
	95% coverage	95%	95%	95.5%
	95% significance	99.9%	99.9%	99.9%
#4b (N~(0, 0.05))	Estimated ΔM and *SE*	0.4931 (0.0985)	0.5051 (0.0996)	0.5703 (0.1072)
	Convergence	100%	100%	100%
	Relative bias ΔM(%)	−1.38	1.02	14.09
	95% coverage	95.7%	95.6%	90.9%
	95% significance	99.9%	99.9%	100%
#4c (N~(0, 0.01))	Estimated ΔM and *SE*	0.4952 (0.0966)	0.5403 (0.0999)	1.4388 (0.2410)
	Convergence	100%	100%	100%
	Relative bias ΔM(%)	−0.96	8.06	187.76
	95% coverage	96.4%	93.6%	3.6%
	95% significance	100%	100%	100%
#4d(N~(0, 0.005))	Estimated ΔM and *SE*	0.4971 (0.0954)	0.5656 (0.0999)	1.9635 (0.2390)
	Convergence	100%	100%	100%
	Relative bias ΔM(%)	−0.58	13.12	292.7
	95% coverage	96.4%	91.4%	0%
	95% significance	100%	100%	100%

When full MI (Model 1) is applied to populations where there are differences on the intercepts between the groups (Pop. 2–7) there is a bias in the latent factor means, which does not occur when applied to a population with no differences (Pop. 1). The only exception is Population 5 with many small intercept differences. However, the coverage is smaller than 95% in this case. When partial MI (Model 2) is applied to populations with intercept differences between all intercepts (Pop. 5–7) there is a large bias, which does not occur when applied to populations with only 2 intercepts having differences between the groups (Pop. 2–4) or without any intercept differences (Pop. 1). Applying approximate MI to all intercepts (Model 3) leads to no bias when applied to a population with no differences (Pop. 1), or a population with small differences (Pop. 5). It does lead to a bias in the other populations with moderate or large intercept differences no matter which prior variance was used (Pop. 2,4,6,7). Applying approximate MI to only those intercepts that are different in the population (Model 4 applied to Pop. 2 and 3) leads to a bias, where the magnitude of the bias is dependent on the prior variance specified.

In population 1, with no intercept differences, the bias is smallest for the Model with strict MI, but the coverage is higher for the models with approximate MI and a high precision of the prior (Models 3c and 3d). In the population with 2 small differences, approximate MI with a high precision of the prior (Models 3c and 3d) modestly outperforms strict and partial MI in terms of bias and coverage. For the populations with moderate and large differences, and invariance on either 2 or 4 items, partial MI is clearly the best model. Also, for the model with many small differences, approximate MI with a high precision of the prior (Models 3c and 3d) just outperforms strict MI and clearly outperforms partial MI. The models with a low precision of the prior were never unbiased.

As pointed out by one of the reviewers, comparing Table 3 and Table 4 on Population 5, partial MI using both ML and Bayes gave smaller relative bias, smaller standard errors, and more accurate 95% coverage than model 3c and model 3d. Indeed, the coverage of model 3c and 3d is too high because in an ideal situation the 95% confidence interval should cover the true parameter value in exactly 95% of the times. The coverage of almost 100% is probably caused by the standard error in model 3c to be overestimated, which can result in the reduction of statistical power. In conclusion, approximate MI should not be applied when full MI holds in the population. If large differences exist in the population on only a few intercepts, partial MI outperforms approximate MI, but partial approximate MI with a large prior variance can also be used. If moderate or small differences exist in the population on only a few intercepts, partial approximate MI is preferred. If small differences exist in the population on many intercepts, approximate MI outperforms applying full MI.

## Resolving the alignment issue

In the previous section we have seen that some of the parameter values, in our case the difference between the latent means, that generated the data are not recovered due to the alignment problem, which reflects an indeterminacy in the CFA. Applying approximate invariance using the DIFF statement tends to pull the deviating parameter toward the average of the parameters across all groups. As a result the deviating parameter will be underestimated and the invariant parameters overestimated, see also the simulation results in Asparouhov and Muthén (2013). With biased intercepts the latent factor means will be biased as well and this is what we call the alignment issue (Asparouhov and Muthén, 2013; in preparation). If one would use plain BSEM the results of the CFA model might be biased in the estimates of the latent mean difference scores, especially when the precision of the DIFF prior is low (i.e., large prior variance), which is undesirable. There are two options to deal with the alignment issues: (1) Freeing the parameters found not invariant (as in Asparouhov and Muthén, 2013), or (2) using the alignment methods available in Mplus v7.1. In the current paper we will focus on the second option, for a comparison of both methods see Asparouhov and Muthén (in preparation for the special issue).

In Mplus v7.1 the alignment-method handles the issue of alignment through rotation. The rotation for the alignment-method uses the same principles as for EFA (Jennrich, 2006) and is described in more details in Asparouhov and Muthén (2013). As stated in the version 7.1 Mplus language addendum (Muthén and Muthén, 2013, p. 2): “the alignment optimization method consists of three steps:

1. Analysis of a configural model with the same number of factors and same pattern of zero factor loadings in all groups.
2. Alignment optimization of the measurement parameters, factor loadings and intercepts/thresholds according to a simplicity criterion that favors few non-invariant measurement parameters.
3. Adjustment of the factor means and variances in line with the optimal alignment.”

The third step in this procedure is expected to decrease the bias in the latent variable means as we discussed above. We included the syntax ANALYSIS: ALIGNMENT = FIXED (BSEM); where FIXED enforces the first latent mean to be zero and the second latent mean to be estimated. When FREE would have been specified all latent means would have been estimated, which is only recommended if more than two groups are specified (Asparouhov and Muthén, 2013. p. 16). Furthermore, BSEM refers to the combination of the alignment-method with the DIFF statements.

To explore the performance of the BSEM-alignment method we ran additional models on population 5 where groups exhibit small differences on the intercepts of all four items (see Table 3). Recall that the bias for this population when applying plain BSEM was 7.36% (*SE* = 0.1353) with the DIFF statement imposed upon all intercepts, but where the factor loadings were constrained across groups (denoted by Model 5a). When population 5 was confronted with a model that imposed plain-BSEM with the DIFF statement on both intercepts and factor loadings (Model 5b) we encountered a bias of 3.62% (*SE* = 0.1279). When the ALIGNMENT = FIXED(BSEM) command was added on top of DIFF statements (Model 5c) the bias appeared to be 4.28% with a lower *SE* of 0.1174. Thus, in this situation the alignment method leads to less bias. Note that these findings are all conditional on normal priors for the DIFF statements with a prior variance of 0.01.

Since prior variance turned out to influence bias and SE's in previous runs we ran Model 5b and Model 5c with prior variances of 0.5 and 0.05 in the DIFF statements. Model 5b with a prior variance of 0.05 yielded a bias of −1.35% (*SE* = 0.2039) and Model 5c yielded a bias of 4.02% (*SE* = 0.1413). Just as with a prior variance of 0.01, if the ALIGNMENT command is added to the DIFF commands, the standard error decreased. When the prior variance of the DIFF statements is increased to 0.5 Model 5b yielded a bias of −43.46% (*SE* = 0.4035), whereas Model 5c with the alignment method performed much better in terms of relative bias and *SE*: −1.34% and a *SE* of 0.1413.

Similar findings were obtained for a population with large differences on all intercepts across groups (Population 7). For this population the bias and SE's were even higher: 399% (*SE* = 0.2011), 391% (*SE* = 0.1940) and 394% (*SE* = 0.3057) for Models 5a, 5b, and 5c with prior variances of 0.01, respectively. It appeared the alignment method, just like plain BSEM, does not resolve the incurred bias when group intercept differences are moderate or high, especially if many items are affected. Since we only used four items in our simulation design more research is needed to investigate whether it is the magnitude of the non-invariance or the number of items affected.

Finally, we ran simulations with models 5a, 5b and 5c for the populations where only two of the items in the population were dissimilar (Populations 1–3, see Table 3), again with a prior variance of 0.01 for the DIFF priors. The results were comparable with the results discussed above (results not shown but these can be requested from the first author). With the ALIGNMENT command we obtained slightly smaller SEs with only small differences in the population compared to approximate MI without the ALIGNMENT command. However, with moderate or large intercept differences between groups the bias and SE for all models were once more too high.

Taken together, DIFF statements imposed upon parameters without the support of an ALIGNMENT command (i.e., plain BSEM) introduced slightly higher standard errors compared to DIFF statements that are combined with the ALIGNMENT command.

## Conclusion

If a researcher wants to compare latent means across groups or over time one has four options:

1. Impose (full or) scalar MI. When a full MI structure results in approporiate model fit any difference in latent means respresents true, unbiased difference between groups/timepoints.
2. Impose partial MI, where one studies the size of the differences between unconstrained loadings and/or intercepts, and constrains all loadings and intercepts except for the one loading/intercept with the largest difference, which is released. If the fit statistics are satisfied, any difference in the latent means is indicative of true mean differences. Sumscores, however, are biased due to the items where differences in the intercepts/factor loadings are allowed (Steinmetz, 2013).
3. Impose no MI, leading to the conclusion that the latent means cannot be used for comparing groups because any difference in the latent means can be caused by many differences.

With Muthén and Asparouhov's introduction of approximate MI (2012a; 2010b; 2013), a fourth option for testing MI became available.

(4a) Approximate MI salvages MI in the case of seemingly ignorable (i.e., near zero) differences between parameters.

Or when combined with partial MI:

(4b) Partial approximate MI, which is a hybrid form of partial MI and approximate MI.

The results of our paper have shown that applying approximate MI might provide a safe passage through the narrow channel between Italy and Sicily in order to facilitate the escape from the mythical sea monsters Scylla and Charybdis, just as Odysseus was able to. The whirlpools caused by Charybdis, who dislikes comparing latent mean scores if the factor loadings/intercepts are dissimilar across groups, can be avoided. The reason is that with approximate MI, parameters are restricted to be closer to each other than with partial MI. The use of Bayesian statistics on the difference in parameters introduces a posterior distribution, which tries to find a compromise between the ideal situation (difference = zero) and the situation we find in the data. The willingness to compromise between ideology and reality has the following effect: the posterior difference in parameters across groups is close enough to its ideal zero to allow latent mean comparisons, yet close enough to the reality of the data to result in acceptable model fit. As was noted by one of the reviewers, a crucial distinction between partial invariance factor models and the Bayesian approach involving priors is that the former typically is coupled with a substantive interpretation of the group differences in the parameters of interest. Although substantive considerations may certainly help inform the nature of group differences, there is always a risk of *ad hoc* reasoning in applications. The Bayesian approach may do more justice to the unexpected and possibly inexplicable failures of invariance. In a related vein, partial measurement models have often been criticized for lacking specificity in the sense that large modification indices of certain items/indicators may actually reflect failures of invariance of other items/indicators (see e.g., Reise et al., 1993).

Likewise it is possible to avoid Scylla, who will devour badly fitting models resulting from forcing scalar MI on a model where differences do exist. Both our empirical example and the simulation study have taught us that there seems to be an optimum specification of the prior variance. The alignment method provides promising results for decreasing the influence of the prior specifications, but more research is warranted.

We recommend the following procedure if the test for full MI fails. First, determine which parameters are different between groups, for example by using modification indices or by using the DIFFERENCE OUTPUT which is obtained when the DIFF statement is used in Mplus. The latter output provides each parameter with a significance test for its deviance with its constrained counterpart. Do not impose MI when there are large parameter differences across groups, or impose approximate MI when you are able to locate just a few deviating parameters. If there are (many) small differences we recommend to apply full approximate MI. Use the ALIGNMENT method when you don't want to use small prior variances in the DIFF statements. We acknowledge the issue of defining “small differences.” With “small” we imply that parameters of substantive interest do not change in a meaningful way if MI does not fully hold (cf. Oberski, 2013). We note that the choice of the priors is extremely important and since the field of approximate MI is rather unexplored we advise always do sensitivity analyses and never just “choose” a prior value. One aspect influencing the definition of a “small difference” is that the prior are sensible for a given data set, and hence, that the choice of the prior variance does have huge implications on the parameter estimates. In particular, because the difference in intercepts is a function of the scaling of the observed variable, as was noted by one of the reviewers, it may be helpful to relate the variances of the normal priors to the scaling (or variability) of the observed variables. For example, for a prior with hyperparameters *N* (0, 0.01) indicates there is a (subjective belief of) 95% chance that the absolute intercept difference is equal or smaller than 0.01 [i.e., sqrt(0.01) = 0.1].

Since the field of approximate MI is relatively new we propose the following research agenda. First, there are two variables influencing the performance of MI: (1) the number of items with differences on the factor loading or intercepts and (2) the size of the difference itself. What we do not know is what the exact cut-off values are for these decisions (number of items and magnitude of differences). This topic needs further attention, given that it can help researchers make informed choices about applying partial or approximate MI without having to test them both. Second, more simulation studies have to be performed to find out which prior specification in which model is to be advised, since the optimum prior specification is model and data dependent. Third, the bias in substantive results if the incorrect type of MI is used should be investigated in more detail. Fourth, more research is needed to study the effects of the alignment method. Fifth, misspecification of the baseline model should be further investigated. A fifth area for further exploration is the comparison of the approximate MI procedure with alternative approaches, for example the commonly used delta-goodness-of-fit-indexes (i.e., ΔGFI; Cheung and Rensvold, 2002; Chen, 2007). And finally, in our simulation study we used a relative large sample size in relation to the degrees of freedom. It should be investigated which sample sizes vis-à-vis model DFs are needed for the Bayesian analysis to work properly. It is expected that the Bayesian test for MI can deal with smaller sample sizes compared to the ML counterparts, as was also the case for regular SEM models, see Lee and Song (2004) and Van de Schoot et al. (submitted).

It should be noted that approximate MI might be an interesting alternative approach for testing MI, but it does not replace the original MI test which is based on, for example chi-square difference testing. Approximate MI, as introduced in our paper provides a first step in this challenging and promising new area of testing and exploring MI if the chi-square test, or any other test, rejects the invariance model. Also, our paper provides a warning not to use approximate MI in all situations where MI is tested, but this warning message also applies to strict MI and partial MI.

### Conflict of interest statement

The authors declare that the research was conducted in the absence of any commercial or financial relationships that could be construed as a potential conflict of interest.

## Footnotes

^1^The two monsters occur in an episode of the adventures of Odysseus; their location is believed to have been at the Strait of Messina between Sicily and the Italian mainland. Scylla, a six-headed sea monster, lived on one end of this strait, while on the other Charybdis resided, causing huge whirlpools. The two monsters were living so close to each other that they created an inescapable threat. Sailors who avoided Charybdis were doomed to meet Scylla and vice versa; it seemed almost impossible to pass the sea strait without being confronted with either of the two mythical monsters.

## Appendix A

### Data:

FILE IS data Tummers.dat;

### Variable:

NAMES ARE CaseNR BCPsych Veran1 Veran2 Veran3 Veran4 mean;

USEVARIABLES ARE Veran1 Veran2 Veran3 Veran4;

MISSING ARE ALL (-9999);

KNOWNCLASS IS g (BCPsych=0 BCPsych=1);

CLASSES IS g(2);

### Analysis:

MODEL IS allfree;

TYPE IS is mixture;

ESTIMATOR IS BAYES;

Bconvergence=0.01;

Biterations = 500000 (20000);

processor is 8;

chains is 8;

bseed 100;

### Model:

%overall%

Willingness by Veran1-Veran4^*^(1-4);

Willingness@1;

[Willingness@0];

[Veran1- Veran4] (nu#_1 - nu#_4);

### Model priors:

DO(1, 4) DIFF(nu1_#-nu2_#) ~ N(0, 0.5);

### Output:

SAMPSTAT TECH1 TECH8 STAND(STDYX);

### Plot:

type is plot2;

## Appendix B

The population input file for the population 3:

### Model population:

f1 by y1@.7 y2@.6 y3@.4 y4@.2;

f1@1; [f1@0];

y1-y4@1;

[y1@0]; [y2@0];

[y3@-.1]; [y4@-.1];

### Model population-g2:

f1 by y1@.7 y2@.6 y3@.4 y4@.2;

f1@1; [f1@.5];

y1-y4@1;

[y1@0]; [y2@0]; [y3@.1]; [y4@.1]
